# Psychological factors associated with trial participation among patients eligible for total knee arthroplasty with a non-surgical treatment arm: a comparative cross-sectional study

**DOI:** 10.2340/17453674.2026.45869

**Published:** 2026-05-08

**Authors:** Nina Jullum KISE, Siri ELIASSEN, Caryl L GAY, Anners LERDAL, Maren Falch LINDBERG, Tor Kjetil NERHUS, Turid ROGNSVÅG, Arild AAMODT, Stig HEIR

**Affiliations:** 1 Department of Orthopedic Surgery, Martina Hansens Hospital, Baerum; 2 Department of Physiotherapy, Martina Hansens Hospital, Baerum, Norway; 3 Department of Family Health Care Nursing, University of California, San Francisco, CA, USA;; 4 Research Department, Lovisenberg Diaconal Hospital, Oslo; 5 Department of Public Health Science and Interdisciplinary Health Sciences, Institute of Health and Society, Faculty of Medicine, University of Oslo; 6 Department of Orthopedic Surgery, Lovisenberg Diaconal Hospital, Oslo; 7 Coastal Hospital in Hagevik, Department of Orthopedic Surgery, Haukeland University Hospital, Bergen, Norway

## Abstract

**Background and purpose:**

Knowledge of psychological factors’ influence on patients’ decisions to participate in randomized controlled trials (RCTs) is scarce. We aimed to compare levels of psychological factors in patients with knee osteoarthritis (OA) eligible for total knee arthroplasty in those who agree to participate in an RCT with those who declined.

**Methods:**

We compared anxiety and depression (Hospital Anxiety and Depression Scale [HADS]), pain-related fear of movement (Fear-Avoidance Belief Questionnaire [FABQ]), catastrophizing (Pain Catastrophizing Scale [PCS]), knee symptoms (Knee Osteoarthritis and Outcome Scale [KOOS]), and knee awareness (Forgotten Joint Score [FJS]), in patients in the Multidisciplinary Intervention in Total Knee Arthroplasty (MultiKnee) trial (n = 280) and in patients who declined (n = 373). Simple and multiple logistic regression models explored associations between psychological factors and patients’ willingness to participate.

**Results:**

Patients declining participation had more fear avoidance (FABQ 1.6 points higher, 95% confidence interval [CI] 0.6–2.6), more knee awareness (FJS 4.0 points lower, CI 1.9–6.1), and worse KOOS scores (ranging from 3.4, CI 0.6–6.1 to 5.6 points, CI 3.1–8.2). In simple regression analysis, each 1-point increase in FABQ-Physical activity score was associated with a 3.9% decrease in odds of participating (OR 0.96, CI 0.94–0.99), and in adjusted analyses, a 3.0% decrease in odds (OR 0.97, CI 0.94–1.0). When considering a clinically meaningful difference of 4 points, this corresponds to a 14.7% reduction in crude odds and a 11.5% reduction in adjusted odds of study participation. Each 1-point increase in HADS Anxiety score was associated with a 5.4% increase in odds of participating (OR 1.05, CI 1.00–1.11). HADS Depression and PCS were not associated with RCT participation.

**Conclusion:**

Patients with higher fear avoidance of physical activity were less willing to participate in the RCT, while patients with higher anxiety were more willing. These findings may weaken the generalizability of the findings from the RCT.

For a randomized controlled trial (RCT) to have external validity, the patients included must be representative of those patients with the same diagnosis who are eligible for the study. The recruitment process must not be biased by excluding certain patient groups with special characteristics or additional diagnoses. Recruitment challenges may be related to the study burden; potential risks associated with the study, the invasiveness of testing, and number of follow-up visits [1]. Furthermore, most patients prefer to receive active treatment rather than be allocated to the control group [2]. According to the OARSI guidelines for osteoarthritis (OA) treatment [3], most patients with knee OA have already undergone non-surgical treatments prior to the general practitioner’s referral for total knee arthroplasty (TKA) and recruiting them into a study including a non-surgical treatment allocation is particularly challenging.

In knee OA, pain is one of the most bothersome symptoms [4,5]. Higher levels of pain, lower physical function, and poorer general health are associated with higher levels of psychological factors like depression and anxiety [6], and pain-related catastrophizing [7,8]. In patients with acute low back pain, higher levels of pain-related fear of movement are associated with higher risk of development of a chronic pain problem [9] , and a similar mechanism could also explain poor outcomes in OA patients. A recent study of patients with knee OA eligible for knee arthroplasty has shown that higher fear avoidance of physical activity and more pain catastrophizing are associated with higher knee awareness and poorer knee-related quality of life [10].

The aim of our study was to compare levels of pain, other symptoms, knee-related quality of life (QoL), knee awareness, and psychological factors (anxiety, depression, pain-related catastrophizing, and pain-related fear of movement) in 2 groups of patients with knee OA deemed eligible for total knee arthroplasty: those who agreed to participate in an RCT with a one-third chance of postponing the surgery by 1 year, and those who declined participation.

We hypothesized that psychological factors are associated with patients’ decisions concerning RCT participation, which might result in selection bias in the RCTs.

## Methods

Our study is a comparative cross-sectional study, and the reporting follows the Reporting of Observational Studies in Epidemiology (STROBE) checklist for cross-sectional studies [11].

The study compares 2 patient groups: 280 patients from the MultiKnee (Multidisciplinary Intervention in Total Knee Arthroplasty) trial and 373 patients from an observational cohort. All patients had knee OA assessed by an orthopedic surgeon as requiring TKA, and all patients fulfilled the inclusion criteria for the MultiKnee trial. Inclusion criteria were age 18–79 years, body mass index (BMI) < 40, radiographic OA grade of 3 or 4 according to Kellgren and Lawrence (KL) [12], American Society of Anesthesiologists Physical Status classification (ASA) grade 1–3, and ability to understand Norwegian. Exclusion criteria were previous total or partial arthroplasty in the index knee, large axis deviations (planned use of a hinged prosthesis), diagnosis of dementia, or diagnosis of a chronic inflammatory joint disease (e.g., Bechterew’s disease or rheumatoid arthritis).

The MultiKnee trial is a multi-center RCT [13], recruiting patients from September 2020 to October 2023 from 3 Norwegian hospitals: Lovisenberg Diaconal Hospital (LDH), the Coast Hospital in Hagevik (CiH), and Martina Hansen’s Hospital (MHH). This 3-armed trial randomized to treatment with education, physiotherapist-assisted exercise therapy, and internet-delivered cognitive behavioral therapy (iCBT), either alone or in combination with TKA compared with a surgery-only control. Patients randomized to the non-surgical group were asked to postpone the operation for at least 12 months.

Patients who fulfilled the inclusion criteria but declined participation in the RCT were asked for the reasons they opted not to participate and to fill in the same baseline patient-reported outcome measures (PROMs) as the included patients; 373 of these patients returned the PROMs. Hence, this study compares the baseline data for the 280 patients in the MultiKnee RCT (hereafter referred to as the “RCT participators”) and the 373 patients in the observational cohort (hereafter referred to as the “RCT decliners”).

The Service for Sensitive Data at the University of Oslo [14] was used to electronically collect and store all patient-reported data, a method that ensures a complete data set without missing items. In addition to demographic characteristics (e.g. age, sex, and BMI), the questionnaires contained reliable and validated PROMs for assessing psychological and clinical factors.

### Psychological questionnaires

The 4 psychological factors assessed were: pain-related catastrophizing with the Pain Catastrophizing Scale (PCS) [15-17], fear avoidance of physical activity with the Fear-Avoidance Belief Questionnaire (FABQ) physical activity subscale [18], and symptoms of anxiety and depression with the 2 subscales of the Hospital Anxiety and Depression Scale (HADS) [19].

The PCS contains 13 items assessing 3 dimensions of catastrophizing (i.e., rumination, magnification, helplessness), and also yields a total score (range 0–52), with higher scores indicating more catastrophizing [15-17]. The FABQ consists of the 2 subscales fear-avoidance beliefs for work and physical activity [18], but only the physical activity subscale was used in this study. Because no minimal clinically important difference (MCID) has been established for the FABQ physical activity scale in patients with knee osteoarthritis, we used an MCID of 4 points based on evidence from patients with chronic low back pain [20]. Higher scores (range 0–24) indicate more avoidance. The HADS consists of 14 items, which constitute an anxiety subscale and a depression subscale. Higher scores (range 0–21) indicate more anxiety or depression. The scores can be dichotomized, scores < 8 suggest no anxiety or depression, and scores > 8 suggest mild to severe anxiety or depression (19). According to Bjelland et al., the Norwegian version of the HADS has excellent psychometric properties [21].

### Measures of knee pain, other symptoms, knee function, and knee awareness

2 questionnaires were used to assess the clinical factors of knee pain, other knee symptoms, knee function, and knee awareness: the Knee Injury and Osteoarthritis Outcome Score (KOOS) [22-24] and the Forgotten Joint Score (FJS) [25].

The Knee Injury and Osteoarthritis Outcome Score (KOOS), a 42-item survey, was used to measure knee pain, other knee-related symptoms, and knee function [22-24]. KOOS has 5 subscales: pain, other symptoms, activities of daily living (ADL), function in sport and recreation, and knee-related quality of life (QoL). The 42 items carry equal weighting (0–4), with higher item scores indicating worse outcomes. For each subscale, the scores are reversed and transformed to a 0–100 scale, with 0 indicating extreme knee problems and 100 indicating no problems [26].

The FJS measures knee awareness, or the patients’ ability to forget about their knees in everyday life [27]. The questionnaire contains 12 statements, and the patients rate their agreement on a scale that ranges from 0 (never) to 4 (mostly). The scores are transformed and reversed to obtain the final score (range 0–100), and higher scores indicate less awareness of the knee. The FJS has good construct validity and test–retest reliability and low ceiling and floor effects and is a valuable tool in discriminating good vs excellent outcomes [28].

### Statistics

IBM SPSS statistical software version 29.0 (IBM Corp, Armonk, NY, USA) was used to analyze the data. Descriptive data is presented as means and standard deviations (SD) for continuous variables or as counts and percentages for categorical variables. We used independent sample t-tests and Pearson’s chi-square tests to compare patients who opted to participate with those who declined by demographic status, pain and knee symptoms, and psychological factors. Effect sizes were calculated for the differences between the groups according to Cohen’s d, defined as small > 0.2; medium > 0.5; large > 0.8. A d-value of > 0.4 was considered to be a clinically meaningful difference [29]. Simple and multiple logistic regression models were used to analyze associations between the independent variables (the psychological factors: anxiety, depression, fear avoidance, and pain catastrophizing) and the dependent variable: participants’ decision to participate in the MultiKnee RCT or not.

Separate multiple regression models were fitted for each independent factor, adjusted for the following possible confounders: age, sex and BMI, as well as level of knee pain, one of the most frequently reported symptoms of knee OA (measured with the KOOS pain subscale).

Continuous HADS subscales were included in the regression models, and dichotomized HADS subscales were used to compare the frequency of anxiety and depression in the 2 patient groups.

Regression coefficients (Exp (B)) were presented as odds ratio (OR) estimates with 95% confidence intervals (CI), and the significance level was set to P = 0.05. The OR estimates indicate change in the odds of participating in the RCT when the independent variable increases by 1 unit. ORs less than 1.0 indicate negative associations and ORs larger than 1.0 indicate positive associations between the dependent and independent variables.

### Ethics, data sharing plan, funding, use of AI, and disclosures

Electronic consent for inclusion was provided by all patients. The MultiKnee trial was performed according to the Declaration of Helsinki, approved by the Ethics Committee of South-Eastern Norway Regional Health Authority (2017/968) and registered in ClinicalTrials.gov (NCT03771430).

Raw data used in this manuscript is available on request to the corresponding author.

Anners Lerdal is funded by the Research Council of Norway (#287816). Maren Falch Lindberg is funded by the South-Eastern Norway Regional Health Authority (#2022007). The MultiKnee trial is supported by the Research Council of Norway (#287816). AI tools were not used.

Each author certifies that he or she has no commercial associations (e.g., consultancies, stock ownership, equity interest, patent/licensing arrangements, etc.) that might pose a conflict of interest in connection with the submitted article. The authors declare no financial or non-financial competing interests. Complete disclosure of interest forms according to ICMJE are available on the article page, doi: 10.2340/17453674.2026.45869

## Results

**Figure 1:**
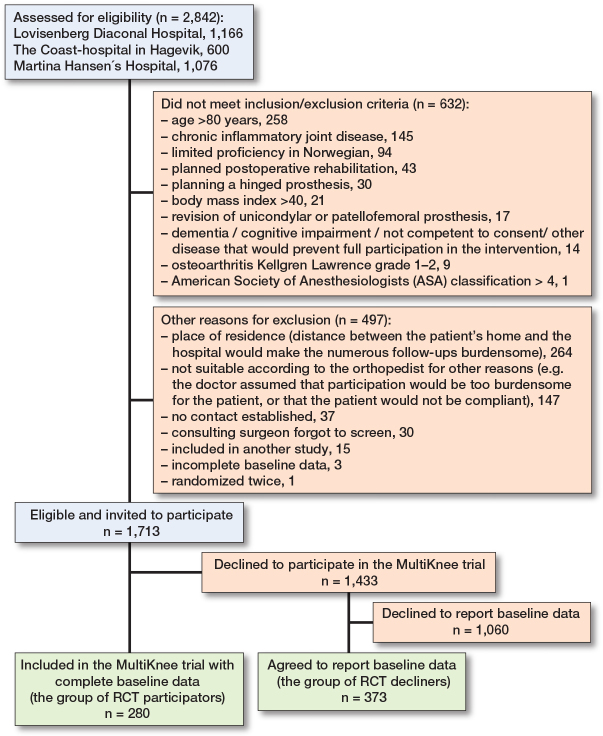
Flowchart of included participants with knee osteoarthritis evaluated to show indication for knee replacement.

The participant flowchart is shown in Figure 1.

This study included 280 patients with a mean age of 67.1 years (SD 7.7), 40% were men, and mean BMI was 28.6 (SD 4.4). There were no differences between the 2 patient groups regarding age or sex, but RCT decliners’ BMI was 0.9 points lower than RCT participators’ (CI 0.2–1.6).

Compared with RCT participators, RCT decliners had more fear avoidance (FABQ 1.6 points higher; CI 0.6–2.6) and more knee awareness (FJS 4.0 points lower; CI 1.9–6.1), both with small-to-medium effect sizes of the difference between the groups (Cohen’s d = 0.3). RCT decliners had worse scores on all KOOS subscales: Symptoms (3.4 points, Cohen’s d = 0.2), Pain (5.6 points, Cohen’s d = 0.3), ADL (4.4 points, Cohen’s d = 0.2), Sport & Recreation (4.2 points Cohen’s d = 0.3), and QoL (4.6 points, Cohen’s d = 0.3) (Table 1).

**Table 1:** Demography, psychological factors, knee pain, and function. Values are count (%) unless otherwise specified

Item	All patients n = 653	RCT participators n = 280	RCT decliners n = 373	Difference between groups (CI) ^a^	Effect size of difference ^b^
Demography					
Age, years **^c^**	67 (7.7)	67 (7.8)	67 (7.6)	–0.4 (–1.6 to 0.7)	–0.1
Sex, male	260 (40)	105 (38)	155 (42)		
Body mass index **^c^**	28.6 (4.4)	29.1 (4.4)	28.2 (4.4)	–0.9 (–1.6 to –0.2) **^d^**	–0.2
Psychological factors					
HADS subscales					
Anxiety **^c^**	3.6 (3.2)	3.8 (3.4)	3.5 (2.9)	–0.3 (–0.8 to 0.2)	–0.1
Depression **^c^**	3.2 (2.7)	3.3 (2.9)	3.1 (2.6)	–0.2 (–0.7 to 0.2)	–0.1
Anxiety < 8	580 (88)	242 (86)	334 (90)		
Anxiety > 8	77 (12)	38 (14)	39 (10)	(P = 0.2	
Depression < 8	599 (91)	253(90)	343 (92)		
Depression > 8	58 (8.8)	27 (9.6)	30 (8.2)	(P = 0.5	
PCS					
Total **^c^**	14.3 (10.6)	13.9 (10.5)	14.6 (10.6)	0.7 (–0.9 to 2.4)	0.1
Rumination **^c^**	4.8 (3.8)	4.7 (3.9)	4.9 (3.8)	0.2 (–0.4 to 0.9)	0.1
Magnification **^c^**	2.4 (2.3)	2.4 (2.2)	2.3 (2.3)	–0.0 (–0.4 to 0.3)	0.0
Helplessness **^c^**	7.1 (5.3)	6.8 (5.3)	7.4 (5.4)	0.6 (–0.3 to 1.4)	0.1
FABQ Physical activity subscale **^c^**	12.6 (6.3)	11.7 (6.4)	13.3 (6.1)	1.6 (0.6 to 2.6) **^d^**	0.3
Knee pain and symptoms					
KOOS subscales					
Symptoms **^c^**	48.8 (18.0)	50.7 (18.2)	47.3 (17.7)	–3.4 (–6.1 to –0.6) **^d^**	–0.2
Pain **^c^**	45.2 (16.7)	48.5 (17.3)	42.8 (15.9)	–5.6 (–8.2 to –3.1) **^d^**	–0.3
ADL **^c^**	55.5 (18.3)	58.0 (18.2)	53.6 (18.2)	–4.4 (–7.2 to –1.6) **^d^**	–0.2
Sport & Recreation **^c^**	16.4 (15.4)	18.8 (16.7)	14.6 (14.0)	–4.2 (–6.6 to –1.8) **^d^**	–0.3
Quality of Life **^c^**	28.1 (13.8)	30.7 (14.2)	26.1 (13.2)	–4.6 (–6.7 to –2.5) **^d^**	–0.3
FJS **^c^**	16.2 (13.6)	18.5 (14.4)	14.5 (12.6)	–4.0 (–6.1 to –1.9) **^d^**	–0.3

a P values are given for the dichotomized HADS subscale.

b Standardized effect size given by Cohen’s d (difference between groups divided by their pooled standard deviation).

c Values are mean (standard deviation).

d Significant.

RCT: Randomized controlled trial; CI: 95% confidence interval.

HADS: Hospital Anxiety and Depression Scale, subscales (range 0–21, higher = more anxiety/depression) and dichotomized (< 8 no anxiety/depression, > 8 moderate to severe anxiety/depression).

PCS: Pain Catastrophizing Scale: Total: range 0–52, higher = more catastrophizing, subscales: Rumination: 0–16, Magnification: 0–12, Helplessness: 0–24.

FABQ: Fear-Avoidance Belief Questionnaire, Physical activity subscale (range 0–24, higher = more avoidance).

KOOS: Knee Injury and Osteoarthritis Outcome Score, (range 0–100, higher = better).

FJS: Forgotten Joint Score, (range 0–100, higher = better).

There were no statistically significant group differences in anxiety (mean HADS score 3.6, SD 3.2) or depression (mean HADS score 3.2, SD 2.7) or the presence of mild to severe anxiety or depression (defined as HADS scores ≥ 8 points), or pain catastrophizing (mean PCS total score 14.3, SD 10.6) (see Table 1).

In simple logistic regression analysis (Table 2), each 1-point increase in FABQ-Physical activity score was associated with a 3.9% decrease in the odds of participating (OR 0.96, CI 0.94–0.99), while in the adjusted analysis, each 1-point increase was associated with a 3.0% decrease in the odds (OR 0.97, CI 0.94–1.0).

**Table 2:** Simple and multiple logistic regression analyses of psychological variables’ associations with the participants’ choice to participate in the RCT or not

Independent variables	Simple logistic regression models		Multiple logistic regression models	
	Odds ratio (CI )	P value	Odds ratio (CI )	P value
HADS Anxiety	1.03 (0.98–1.08)	0.2	1.05 (1.00–1.11)	< 0.05
HADS Depression	1.03 (0.97–1.09)	0.3	1.06 (0.99–1.12)	0.08
PCS Total score	0.99 (0.98–1.01)	0.4	1.00 (0.99–1.02)	0.6
PCS Rumination	0.99 (0.95–1.03)	0.5	1.01 (0.97–1.06)	0.7
PCS Magnification	1.01 (0.94–1.08)	0.8	1.05 (0.98–1.13)	0.2
PCS Helplessness	0.98 (0.95–1.01)	0.2	1.00 (0.97–1.04)	0.9
FABQ Physical activity scale	0.96 (0.94–0.99)	0.002	0.97 (0.94–1.00)	0.02

The dependent variable is the participants’ decision to participate in the RCT or not.

The multiple logistic regression models control for age, sex, BMI, and KOOS-Pain.

For abbreviations, see Table 1.

In the multiple logistic regression analysis controlling for age, sex, BMI, and KOOS-Pain, each 1-point increase in HADS-Anxiety score was associated with a 5.4% increase in the odds of participating (OR 1.05; CI 1.00–1.11). HADS-Depression and PCS were not associated with RCT participation.

The 373 patients in the RCT decliner group reported a total of 586 answers to the question of why they chose to decline RCT participation, and the most frequent reasons were “because I risk postponing the operation” and “because I have already gone through physiotherapy,” given by 71% and 41% of the sample, respectively. Only 1.9% and 2.1% reported “risk of going through cognitive therapy” or “risk of going through physiotherapist-guided exercise therapy” as reasons for declining (Table 3).

**Table 3:** Reasons for not participating in the RCT given by participants in the group of RCT decliners (n = 373). Values are number of answers and percentage of cohort

Why did you choose not to participate? ^a^	n (%)
I risk postponing my TKA surgery	268 (71)
I have already gone through physiotherapist-guided exercise therapy	155 (41)
The study visits would require too much travel	67 (18)
The study would require too much time	37 (9.8)
I want to decide my own treatment	22 (5.8)
I have trouble with electronic devices	16 (4.2)
I risk going through cognitive therapy	8 (2.1)
I risk going through physiotherapist-guided exercise therapy	7 (1.9)
I do not want to expose myself to the Coronavirus	6 (1.6)
Total number of answers	586

a Patients were free to give more than 1 answer.

## Discussion

This is the first study focusing on psychological factors’ influence on the willingness of patients with knee OA to participate in RCTs, especially RCTs that include surgical interventions as well as non-surgical treatments: education, exercise therapy, and cognitive therapy. We showed that patients with increased fear avoidance of physical activity had lower odds of participation in the RCT, that a 1-point increase in the FABQ score was associated with a 3.9% decrease in the odds of participating, and that KOOS-Pain, the unit-based decrease in the odds of participating, was 3.0%.

Although this effect might seem small, the FABQ scale spans a wide range from 0 to 24, and a 1-point score increase is quite modest. When considering a clinically meaningful difference of 4 points, this corresponds to a 14.7% reduction in crude odds and a 11.5% reduction in adjusted odds of study participation.

Further, analyses found no association between depression or pain-related catastrophizing and participation in the RCT, but, in adjusted analyses, a 1-point increase in the HADS Anxiety score was associated with a 5.4% increase in the odds of RCT participation.

The finding that higher fear avoidance of physical activity was associated with decreased participation is in line with the fact that these patients also had more knee symptoms and more knee awareness (3.4–5.7 points worse scores on all KOOS subscales and on FJS). It is meaningful to assume that these patients were more skeptical about being randomized to a treatment group with exercise therapy and that they did not want to risk postponing TKA. In fact, 71% of the patients in the RCT decliner group gave the reason “because I risk postponing the operation” for not participating in the RCT (see Table 3). Hence, the skepticism of patients with knee OA regarding non-surgical treatments is even higher than in a study of ophthalmological patients, where 49% declined inclusion in the RCT because they “were satisfied with the standard care” [30].

Association between general anxiety and increased RCT participation was seen in the crude analysis, but not in the adjusted analysis. The tendency, however, for higher general anxiety levels to possibly be associated with increased participation may seem surprising. One explanation might be that these patients also experience higher levels of anxiety related directly to the surgical procedure. Following detailed information on the non-surgical treatment alternatives of exercise therapy and cognitive therapy, these patients might consider participating in the RCT as an option to postpone or even cancel the surgery.

The physical, psychological, and financial burdens on patients from participating in RCTs is well known [31] and largely explains the patients’ decision not to participate. Recruiting patients to RCTs that compare surgical with non-surgical treatments has previously been described as particularly difficult, as reported in numerous prior studies. For example, from studies of patients with degenerative meniscal tears, the precursor of knee OA [32]: in a study of 140 patients with degenerative menisci comparing arthroscopic partial meniscectomy with exercise therapy, 2.4 patients were screened to include 1 patient [33]. In another study, comparing arthroscopic partial meniscectomy with sham (placebo) surgery, recruiting was even more difficult: as many as 11.9 patients were screened to include 1 patient [34]. In the MultiKnee trial, we screened 2,842 patients to include 280 in the RCT, so 10.2 patients were screened to include 1 patient in the RCT. Hence, it seems as if recruiting patients into a trial with exercise therapy and cognitive therapy as alternatives to surgery is as challenging as recruiting to a trial with sham surgery.

Our cross-sectional comparative study of patients eligible for TKA revealed that patients with more pain-related fear avoidance of physical activity, more knee symptoms, and more knee awareness were less willing to participate, while higher anxiety levels tended to be associated with higher willingness to participate.

Our findings indicate a risk of inclusion bias in studies testing exercise and CBT interventions, such as the MultiKnee trial. These findings need to be taken into account when planning similar studies, both to ensure a balanced sample of patients, and to ensure that factors characterizing the non-participants are assessed so they can be taken into account when analyzing follow-up results in the trial.

Importantly, the observation that patients with higher fear avoidance and more knee symptoms and knee awareness are less willing to be enrolled in an RCT where one of the arms involves exercise without surgery may provide useful knowledge for clinical practice. When caring for patients with knee OA, clinicians should recognize that psychological factors may influence a patient’s willingness and ability to engage in treatments such as exercise therapy, to a similar extent as physiological symptoms. Routine mapping of relevant psychological factors might give clinicians valuable information that can help to suggest alternative or complementary treatment interventions to the patient before undergoing TKA surgery. For example, patients might need additional support to engage in and benefit from exercise therapy if they have high fear avoidance of physical activity. Cognitive therapy has been shown to be a supportive alternative [35].

The challenge is how to efficiently map patients in clinical practice, where limited resources are a common occurrence. Researchers must continue to address these questions and challenges in order to develop shorter and more precise measurement instruments that are suitable for clinical use.

### Strength

A major strength of this comparative cross-sectional study of patients with knee OA eligible for knee TKAs is the structured inclusion of a large sample size. The patients were recruited from the 3 largest knee centers in Norway across 2 health regions, which increases the representativeness of the sample. Further, the study has full datasets with no missing data, due to the use of internet-based electronic data collection, which allows participants to submit forms only when all items are filled in. Both the generic and disease-specific questionnaires used are reliable and validated.

### Limitations

Of the 2,842 screened patients, we have data only from the 280 patients in the RCT sample and 373 in the observational sample. Of the 1,713 patients that met the inclusion and exclusion criteria, we do not know why 1,433 (84%) declined to participate in the RCT, and of those who declined the RCT, 1,060 (74%) also declined to participate in the observational sample. We do not know anything about their psychological status or their levels of knee symptoms and knee awareness. Hence, we were unable to evaluate the nature of possible responder bias.

The relatively small effect sizes of the reported associations (FABQ Physical activity scale OR for participation 0.96–0.97 and HADS Anxiety 1.05 with 95% confidence intervals close to null) means that there is no evidence that these associations translate into clinically meaningful differences in participation behavior.

Because the MCID for the FABQ not has been explored for patients with knee OA, we used the value of an MCID of 4 points found in patients with chronic low back pain, without knowing whether there are significant distinctions between the MCIDs in the 2 patient groups.

Although the FJS is a validated questionnaire in patients with OA, the Norwegian version used in this study has not yet been validated, although it is increasingly used in clinical practice. Hence, it is possible that psychometric properties are not equal to the validated versions.

### Conclusions

The TKA-eligible patients who declined participation in the RCT but accepted inclusion in the observational cohort (26%) had more fear avoidance, worse knee pain, more symptoms, and more knee awareness than the RCT participants. Controlling for other factors, patients with higher anxiety levels were more willing to participate, while patients with pain-related fear avoidance of physical activity were less willing.

*In perspective,* psychological factors’ potential influence on patients’ willingness and ability to actively participate in alternative or complementary treatments warrants further research. There is also a specific need for short and precise measuring instruments to capture relevant psychological risk factors that can be used both in research and in clinical practice.

## Notes

NJK, AL, MFL, TKN, AA, and SH conceived and designed the study. NJK, SE, TKN, AA, SH, and TR collected the data. NJK and CLG performed the statistical analyses. NJK drafted the manuscript. All authors had access to all data, participated in analysis and interpretation of the data, revision of the manuscript, and gave final approval of the version to be published.

Handling co-editors: Taco Gosens and Robin Christensen

*Acta* thanks Troels Christensen and for help with peer review of this manuscript.

The authors would like to thank the patients in this study for their participation. Further, they wish to acknowledge Lovisenberg Diaconal Hospital, the Coastal Hospital in Hagevik, and Martina Hansens Hospital for accessibility to the outpatient and physiotherapy clinics.

This paper is a product stemming from the Norwegian research project “The MultiKnee trial.” Dr Maren Falch Lindberg is the Principal Investigator (PI) and Drs Anners Lerdal and Arild Aamodt are Co-PIs. Ingvild Buset Bergvad, Alexander Eikrem-Lüthi, and Turid Rognsvåg are PhD students supervised by the seniors Drs Anners Lerdal, Jon Magnussen, Maren Falch Lindberg, Mona Badawy, Ove Furnes, and Søren T. Skou. The other members of the MultiKnee research team are Drs Caryl L. Gay, Stig Heir, Inger Holm, Nina Jullum Kise, Tor Kjetil Nerhus, Milada C. Småstuen, and Jan Stubberud. Katrine Rutledal, Deputy Director for Lovisenberg User Board, provided user participation.
